# The Role of lncRNAs in Regulating the Intestinal Mucosal Mechanical Barrier

**DOI:** 10.1155/2021/2294942

**Published:** 2021-11-15

**Authors:** Shanshan Chen, Chi Zhang, Beihui He, Ruonan He, Li Xu, Shuo Zhang

**Affiliations:** The First Affiliated Hospital of Zhejiang Chinese Medical University, Hangzhou, China

## Abstract

lncRNA is a transcript that is more than 200 bp in length. Currently, evidence has shown that lncRNA is of great significance in cell activity, involved in epigenetics, gene transcription, chromatin regulation, etc. The existence of an intestinal mucosal mechanical barrier hinders the invasion of pathogenic bacteria and toxins, maintaining the stability of the intestinal environment. Serious destruction or dysfunction of the mechanical barrier often leads to intestinal diseases. This review first summarizes the ability of lncRNAs to regulate the intestinal mucosal mechanical barrier. We then discussed how lncRNAs participate in various intestinal diseases by regulating the intestinal mucosal mechanical barrier. Finally, we envision its potential as a new marker for diagnosing and treating intestinal inflammatory diseases.

## 1. Introduction

Previous studies have found that although there are as many as 3 billion base pairs in the human genome, few can encode proteins, yet most of them produce a class of RNA, noncoding RNA (ncRNA). It cannot encode proteins. This type of RNA is rich in species, including microRNAs (miRNAs), small nucleolar RNAs (SnoRNAs), lncRNAs, and circRNAs [1]. Among them are lncRNA transcripts longer than 200 bp but do not encode proteins. lncRNA is a by-product of RNA polymerase II (Pol II), with a methyl guanosine cap at its 5′ end and a poly tail at its 3′ segment [2]. Compared to other categories of ncRNAs, lncRNAs show a surprisingly wide range of size, shape, and functionality. These characteristics give them the functional potential that cannot be underestimated [3]. The development of third-generation sequencing technology has expanded and improved the existing lncRNA annotations rapidly, economically, and effectively so that an increasing number of new lncRNAs have been discovered and annotated [4]. lncRNAs are usually located in the nucleus or cytoplasm. They can regulate chromatin and assemble membranous nucleosomes through interactions with other genetic materials such as DNA, RNA, and proteins. Furthermore, they can also change the stability and translation of cytoplasmic mRNAs and interfere with signaling pathways [5]. The rich function of lncRNAs affects gene expression in many physiological and pathological processes. Studies have confirmed that they are involved in neuronal diseases [6], immune response [7], and cancer [8]. Recent findings have shown that lncRNAs play an essential role in intestinal diseases, such as inflammatory bowel disease (IBD), and can potentially be used as biomarkers and therapeutic targets.

More than 10 trillion bacteria, fungi, viruses, and other microorganisms are present in the intestinal mucosa [9]. The intestinal mucosal mechanical barrier prevents these uninvited guests from invading the internal environment. It maintains the dynamic balance between these intestinal florae and intestinal epithelial cells. Moreover, it is the transportation carrier between the body and nutrients, water, and waste [10]. The intestinal mucosal mechanical barrier is the most important part of the intestinal mucosal barrier, which is essentially a defensive layer composed of intestinal mucosal epithelial cells and tight junctions between cells and the bacterial membrane. Its existence can effectively prevent intestinal mucosal injury [11]. Pathophysiological changes, including trauma, local ischemia, total parenteral nutrition (TPN), and intestinal obstruction, can cause acute or chronic damage to the intestinal mucosal barrier [12]. It has been confirmed that intestinal mucosal barrier injury can increase intestinal permeability, usually accompanied by critical diseases, including septicemia and multiple organ failure. This injury may result in high morbidity and mortality rates [13].

Therefore, the identification and characterization of early biomarkers for intestinal inflammatory diseases have become a priority. This review discusses the role of lncRNAs in regulating the mechanical barrier and how they coregulate each other and their target genes.

## 2. Intestinal Mucosal Mechanical Barrier

**Figure 1:**
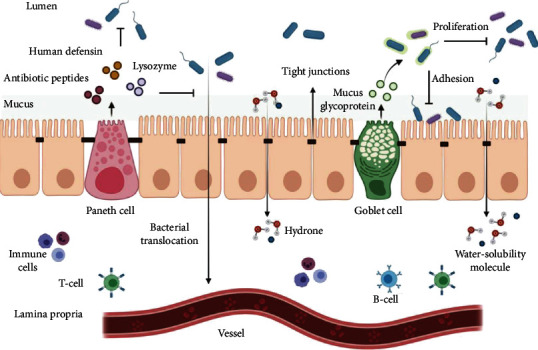
The tight junction between intestinal mucosal epithelial cells and adjacent cells constitute the intestinal mucosal mechanical barrier jointly, which prevents intestinal microorganisms and foreign pathogens from entering the intestinal environment and prevents the occurrence of inflammation.

The mechanical barrier of the intestinal mucosa consists of intestinal epithelial cells and tight junctions between cells and the bacterial membrane. Intestinal epithelial cells contain absorptive cells, Paneth cells, and goblet cells (Figure 1). Intestinal epithelial cells are phagocytosed by bacteria. Paneth cells can secrete lysozyme, natural antibiotic peptides, human defensin 5, and human defensin 6. With the deepening of research, the role of Pan's cells in inhibiting bacterial translocation and preventing enterogenous infection has been gradually explored [14]. Goblet cells secrete mucus glycoproteins, preventing digestive enzymes and harmful substances from damaging epithelial cells in the digestive tract. It can wrap bacteria and compete with pathogenic microorganisms to inhibit adhesin receptors on intestinal epithelial cells. It inhibits bacterial adhesion and colonization in the intestine, thereby restraining the proliferation of intestinal bacteria and intestinal infection [15]. Intercellular junctions are composed of tight junctions, adhesive junctions, gap junctions, and desmosome junctions, of which tight junctions are the core [16]. The tight junctions between adjacent cells are tightly arranged by tight junction protein particles. There are many kinds of proteins, including claudin, occludin, junctional adhesive molecule (JAM), and zonula occludens (ZO) [17]. When there are more than two forms of intercellular junctions between the sides of the adjacent intestinal epithelial cells, we can call it the tight junction complex. The gap between the complexes is so narrow that only water molecules and small molecular water-soluble substances can selectively pass through [18]. The tight junction between cells is similar to an iron fence, which closes the gap between intestinal epithelial cells. It can prevent foreign pathogens from entering the lamina propria, so as to prevent the activation of immune cells in the lamina propria, avoiding the occurrence of abnormal mucosal immune reactions [19]. From a macropoint of view, intestinal motor function is also part of the intestinal mechanical barrier, which prevents the attachment of intestinal bacteria and promotes the self-cleaning function of the intestinal tract [20].

## 3. Regulatory Effects of Different lncRNAs on Intestinal Mucosal Mechanical Barrier

### 3.1. lncRNA H19

lncRNA H19, located in the endometric region 11P15.5 of the human chromosome, is a long noncoding RNA with a length of 2.3 kb, first discovered in 1991 [21]. Its expression reaches its highest level in the human embryo and decreases with aging [22]. Previous studies have found that lncRNAH19 plays a role in physiological and pathological processes such as inflammation, aging, and tumorigenesis [23, 24]. In recent years, an increasing number of studies have reported that it can regulate the intestinal mucosal mechanical barrier through various mechanisms and indirectly participate in the progression of intestinal diseases.

#### 3.1.1. Regulation of Intestinal Mucosal Epithelial Cell Function and Tight Junction by lncRNA H19

Yu et al. [25] suggested that the defense function of Paneth cells and goblet cells in the intestinal mucosal mechanical barrier was enhanced if the H19 gene was specifically knocked out in mice. Autophagy of the intestinal mucosa was also improved. On the contrary, the overexpression of H19 significantly inhibited the functions of Paneth cells and goblet cells, and the autophagy of intestinal mucosa for self-renewal was also significantly weakened, possibly leading to damage to the intestinal mucosa mechanical barrier. Previous studies have found that the RNA-binding protein HUR can effectively regulate the intestinal mucosal mechanical barrier by binding directly to lncRNA H19 [26]. Zou et al. [27] suggested that H19 could indirectly destroy the tight junction of the intestinal mechanical barrier by increasing the expression of miR-675, destroying the structure of ZO-1 and E-cadherin in tight junction proteins, and inhibiting their translation. By increasing the content of HUR, which competes for the binding of H19 and whose effect on miR-675 was weakened, the upregulated expression of ZO-1 and E-cadherin could be detected, and the function of the intestinal mucosal mechanical barrier gradually recovered. The authors suggest that H19 can destroy the defense function of the intestinal mucosal mechanical barrier by increasing the expression level of downstream miR-675. These two studies illustrate that H19 can destroy the intestinal mucosal mechanical barrier. However, many studies have found that H19 has an opposite effect on the intestinal barrier through some mechanisms. Li et al. [28] found that intestinal autophagy was activated after severe burn using a mouse model; this could increase the transcription level of lncRNA H19 and suggested that lncRNA H19 might regulate the repair of EGF after intestinal mucosal injury after burn through miRNA LET-7G. Another study verified this conjecture and found that the autophagy-mediated H19 expression increased in the intestine of severely burned mice and acted as a sponge combined with let-7 g to regulate EGF, suggesting that H19 may be a therapeutic target and biomarker of intestinal mucosal injury after burn [29]. In addition, H19 can also regulate the intestinal mucosal mechanical barrier by regulating the expression of AQPs. Aquaporin (AQP) is a small (30 kDa/monomer) hydrophobic membrane integrin belonging to the special superfamily membrane integrin of MIP (main intrinsic protein). AQPs are responsible for transporting liquids and electrolytes [30]. AQP3 mainly exists in human intestinal epithelial cells. Because of its existence, the intestinal mucosa can reverse the osmotic gradient to complete the absorption and elimination of water to achieve the normal function of the intestine and the balance of human body fluid. Zhang et al. [31] found that cell permeability increased significantly after the knockout of AQP3 in a Caco-2 cell line, which may be related to the inhibition of tight junction proteins in the intestinal mucosal mechanical barrier, but the specific mechanism is still unclear. In addition, Chao et al. [32] found that compared to healthy colon tissue, the expression of lncRNA H19, AQP1, and AQP3 in the colon tissue of patients with IBS-D decreased. The author further demonstrated that the expression of lncRNA H19 was positively correlated with AQP1 and AQP3. This experiment revealed that lncRNA H19 might participate in developing IBS-D by regulating the intestinal mucosal mechanical barrier. Geng et al. [33] detected the level of lncRNAH19 in intestinal tissues of IBD mice and humans and found that its expression level was significantly higher than that in normal tissues. H19 may promote the proliferation of intestinal epithelial cells and repair inflammatory mucosa by inhibiting the expression of p53 protein, microRNA34a, and let-7 and promote IEC proliferation and epithelial regeneration.

#### 3.1.2. Inhibiting the Expression of Vitamin D Receptor (VDR)

VDR is a nucleophilic protein that can mediate 1,25(OH) D and exerts its biological effects. In recent years, many studies have reported the involvement of VDR in the process of ulcerative colitis (UC) and other diseases [34]. Furthermore, several studies have shown that 1,25(OH)_2_D_3_ can effectively prevent intestinal mucosal mechanical barrier damage [35]. Chen et al. [36] detected the expression levels of H19, miR-675-5p, and VDR in 12 patients with UC. The results showed that, compared to normal tissues, the expression of VDR was significantly downregulated in patients with UC, while the expression of H19 was significantly increased. In an experimental study of the Caco-2 cell line, it was found that overexpressed lncRNA H19 could inhibit the expression of VDR by upregulating miR-675-5p. Yet, this effect could be partially weakened by the miR-675-5p inhibitor. Based on this, the authors inferred that lncRNA H19 could inhibit the expression of VDR and damage the intestinal mucosal mechanical barrier. At the same time, miR-675-5p mentioned in the experiment can only partially affect the effect of lncRNA H19. Therefore, the research on VDR mentioned above still needs to be further improved.

### 3.2. CCAT1-lncRNA

CCAT1-lncRNA, also known as colon cancer-associated transcript-1, was first found in colon cancer with a length of 2628 nucleotides and located on chromosome 8q24.2 [37]. CCAT1 is highly elevated in many types of cancers such as lung adenocarcinoma, gastric cancer, colorectal cancer, and esophageal squamous cell carcinoma and plays an important role in many biological processes, such as invasion, proliferation, drug resistance, migration, and survival [38–42]. In the last few years, some studies have suggested that CCAT1 can inhibit the function of the intestinal mucosal mechanical barrier through some mechanisms, leading to intestinal diseases. The mechanism of CCAT1-induced malignant transformation of IBD into CRC has also been discovered for the first time. Ma and other studies have found that CCAT1 can promote high levels of myosin light chain kinase (MLCK) expression by acting as a molecular sponge of miRNA and competitively binding to miR-185-3p, leading to an increase in intestinal barrier permeability and the weakening of intestinal mucosal barrier function in patients with IBD, resulting in malignant diseases [43].

### 3.3. PlncRNA1

PlncRNA1, also called CBR3AS1, located in the antisense region of carbonyl reductase 3 (CBR3), was first found to be upregulated in prostate cancer [44]. It has been confirmed that it is also associated with other types of cancer, such as retinoblastoma, colorectal, and liver cancers [45–47]. A recent study suggested that PlncRNA1 can regulate the expression of downstream miR-34c by cooperating with the Myc gene, indirectly enhancing the expression of zinc finger protein (MAZ), ZO-1, and occludin in tight junction proteins and enhance the mechanical barrier of the intestinal mucosa. The authors confirmed that the overexpression of PlncRNA1 enhances the defense of the intestinal mucosal barrier against sodium sulfate glucose (DSS) injury. The authors concluded that PlncRNA1 could regulate the tight junction protein of the intestinal mucosal mechanical barrier and enhance the defense function of the intestinal barrier by regulating the content of downstream miR-34c [48].

### 3.4. lncRNA neat1

lncRNA neat1 is a nuclear-rich lncRNA located in accessory plaques [49], the integrity of accessory plaques [50]. Presently, studies have found that it is highly upregulated or downregulated in various tumor entities. Its main role is as a competitive endogenous RNA (Cerna) and competitive binding of tumor suppressor microRNA (miRNA). Sponge miRNA loses the ability to degrade, silence, or hinder its downstream, mainly carcinogenic-targeted transcript translation, and finally promotes cancer occurrence [51]. A recent study detected lncRNA NEAT1 in the intestinal mucosa of patients with IBD and found that its expression level was significantly upregulated compared to that in normal tissues. After further study, the authors found that specific knockout of NEAT1 in IBD mice could significantly reduce abnormally increased intestinal permeability, mediate macrophage polarization through the exocrine pathway, and weaken intestinal inflammation. Finally, the authors concluded that lncRNA NEAT1 could increase intestinal permeability abnormally and promote the inflammatory response in IBD by destroying the intestinal mucosal mechanical barrier [52].

### 3.5. lncRNA SPRY4-IT1

lncRNA SPRY4-IT1 is a 706 bp-length transcript found first in a sequence of adipose tissue cDNA [53]. It has been further confirmed to be widely expressed in various human tissues, including the intestinal mucosa [54]. SPRY4-IT1 is transcribed from the SPRY4 gene in the intron region, but SPRY4-IT1 is completely different in structure from SPRY4mRNA [55]. Previous studies have found that SPRY4-IT1 is highly expressed in various cancers, including melanoma [54], colorectal cancer [56], breast cancer [57], and systemic scleroderma [58]. Currently, some studies have found that the lncRNA SPRY4-IT can regulate the intestinal epithelial barrier. Xiao et al. [59] showed that lncRNA SPRY4-IT1 regulates intestinal epithelial barrier function by interacting with HUR and changing the expression of tight junction (TJ) proteins. In the in vitro experiment, the authors downregulated the expression of SPRY4-IT1. They found that the expression of TJ in the intestinal mucosal mechanical barrier was significantly inhibited, impairing the defense function of the intestinal barrier. On the contrary, increasing the expression level of SPRY4-IT1 in the intestinal mucosa not only prevented TJ inhibition induced by cecal ligation and perforation (CIP) but also protected the intestinal epithelial barrier from septic stress in vivo. The authors believe that SPRY4-IT1 is essential for maintaining the function of the intestinal mucosal mechanical barrier. Although it cannot increase the basic level of TJ proteins, it promotes the expression of tight junction proteins.

### 3.6. lncRNA uc.173

lncRNA (T-UCR), transcribed from UCR, originates from genomic elements located in many mammalian genomes, which is quite conservative in evolution; hence, it nickname, “dark matter” (DarkMaterial) in the human genome [60]. With the deepening of the study, researchers have found that it positively affects the intestinal mucosal mechanical barrier. Xiao et al. [61] proposed that uc.173 downregulates the expression of miRNA195 in intestinal epithelial cells by destroying the stability of pri-miR195. They found that miRNA195 can inhibit the expression of many proteins (CDK4, CDK6, CCND1, STIM1, and WEE1), which are important for cell migration and proliferation and significantly hinder the renewal of the intestinal mucosal barrier. In summary, the authors drew the following conclusions: in the analysis of intestinal epithelial cells and mice, uc.173 noncoding RNAs regulate the intestinal mucosal barrier and stimulate intestinal epithelial renewal by reducing the abundance of miRNA195. Wang et al. [62] found that uc.173 can act as a natural bait for miR-29b, specifically binding to RNA, reducing its inhibitory effect on CLDN1mRNA, and promoting the translation of the tight junction protein claudin-1 (CLDN1) and the repair of the intestinal mucosal mechanical barrier.

### 3.7. lncRNA Bmp1

Mouse Bmp1 is a full-length 4464 bp gene located on chromosome 14qD2. It was first found in bones and has been reported to play a variety of functions in bone formation [63]. Some studies have also found that this may be related to susceptibility to lung cancer [64]. Presently, some studies have found that the expression of its transcriptional product lncRNABmp1 is significantly increased in a burn mouse model, and the Bmp1 content is upregulated after intestinal mucosal injury. Through the experiment, the authors concluded that Bmp1 overexpression has a protective effect on the intestine of scalded mice, which can significantly improve the proliferation and migration of IEC-6 or HIEC-6 cells of intestinal crypt epithelial cells in rats through the Bmp1/miR-128-3p/PHF6/PI3K/AKT pathway and promote the repair of intestinal mucosal mechanical barrier [65]. This overexpression of lncRNA BMP1 can promote the repair of the intestinal mucosal barrier in burn patients.

### 3.8. lncRNA BC012900

In 2016, a study found for the first time that IBD is related to lncRNA, which is regulated by inflammatory stimulation and plays an important role in intestinal epithelial function. Wu et al. [66] screened 17,000 lncRNA genomes. By detecting lncRNA microarray and quantitative RT-PCR, the authors found that compared to normal tissues, 455 lncRNAs were significantly differentially expressed in the colon tissues of patients with UC in the active stage, among which lncRNA BC012900 was selected. This study found that overexpression of BC012900 downregulated the proliferation of HCT116 and HT29 intestinal epithelial cell lines and increased the susceptibility of intestinal epithelial cells to apoptosis, which was reversed by knocking out BC012900 expression by siRNA. Finally, the author concluded that overexpression of lncRNABC012900 in epithelial cells could significantly inhibit cell proliferation and increase cell sensitivity to apoptosis (Table 1).

## 4. lncRNA Participates in the Development of Intestinal Diseases by Regulating the Intestinal Mucosal Mechanical Barrier

### 4.1. lncRNA Participates in the Occurrence and Development of IBD by Regulating Intestinal Mucosal Mechanical Barrier

#### 4.1.1. The Change of Intestinal Mucosal Permeability Is an Important Prodromal Symptom of IBD

Two new studies in 2020 have shown that changes in intestinal epithelial permeability in patients with IBD precede the onset of clinical symptoms, suggesting that specific interventions can be adopted to prevent IBD in the early stages of the disease [67]. Turpin et al. [68] reported a 7-year study on asymptomatic first-degree relatives of 1420 patients with Crohn's disease (CD) and quantified the permeability of the intestinal mucosal mechanical barrier by the ratio of lactulose to mannitol excretion fraction (LMR). It was found that intestinal permeability in patients with Crohn's disease was significantly higher during the follow-up period than in healthy people without Crohn's disease. The second study conducted by Torres et al. [69] examined serum samples from the Defense Department's Serum Bank (DoDSR), including Crohn's disease, ulcerative colitis (UC), and healthy individuals, and obtained a group of 51 protein biomarkers through data analysis. An IBD prediction model including hypothetical predictors (specific predictors cannot be determined) and protein biomarkers was established. When the AUROC is less than or equal to 0.76, the patient can predict the occurrence of Crohn's disease within 5 years; when the AUROC is less than or equal to 0.87, the patient can be diagnosed with Crohn's disease within one year at the earliest. These two studies suggest that the intestinal barrier function of patients with IBD has been disturbed a few years before clinical symptoms appeared, and the detection of intestinal permeability can alert us in advance of IBD occurrence.

#### 4.1.2. The Injury of Intestinal Mucosal Mechanical Barrier Is a Typical Manifestation of IBD

By observing the lesion site of UC and CD patients, it was found that the tight junction structure of the lesion mucosa was most obviously destroyed [70], and the expression of occlusive tight junction protein was significantly downregulated, while the expression of pore-like tight junction protein was increased. These processes are often positively correlated with IBD symptoms [71]. IBD also induces intestinal barrier damage by inducing epithelial cell death or apoptosis. UC patients and colitis mouse models were accompanied by apparent death and destruction of colonic epithelial cells; through the anatomy of these organizations, there are many crypt-like microabscesses composed of inflammatory cells and dead cells in the intestinal tract of IBD mice, which increases the permeability of intestinal mucosal mechanical barrier in mice [72].

**Figure 2:**
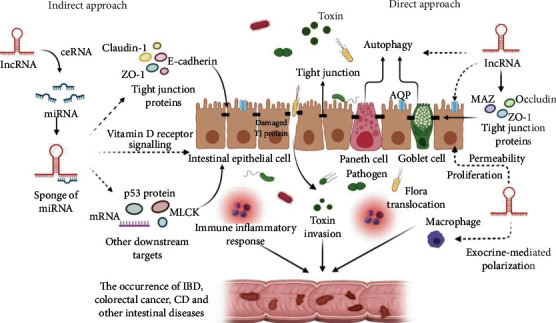
lncRNAs regulate the intestinal mucosal barrier directly (directly regulating TJ protein or AQPs) or indirectly (through miRNAs or other intermediates). The destruction of intestinal mucosal barrier participates in the progression of intestinal diseases such as IBD, colorectal cancer, and celiac disease through immune inflammatory reaction, toxin invasion, and flora translocation.

In the past few years, an increasing number of studies have shown that lncRNAs are closely involved in the pathogenesis of IBD [73]. lncRNAs are involved in many processes of IBD, such as the regulation of intestinal epithelial cell apoptosis and intercellular tight junction proteins related to lipid metabolism (Figure 2), thus regulating the permeability of the intestinal mucosal mechanical barrier [74]. Recent studies have reported that lncRNA CNN3-206 expression is increased in the intestinal tissue of CD patients. By acting as a molecular sponge to adsorb miR-212, activating the lncRNA CNN3-206-miR-212-Caspase10 regulatory network leads to increased apoptosis, migration, and invasion of intestinal epithelial cells [75]. Yang et al. [76] reported a new lncRNA named CRNDE, which can regulate the expression of downstream miR-495 and SOCS1, indirectly induce apoptosis of intestinal mucosal epithelial cells and aggravate the inflammatory response in IBD.

### 4.2. lncRNA Participates in Colorectal Cancer by Regulating Intestinal Mucosal Mechanical Barrier

Intestinal mucosal mechanical barrier deficiency can lead to direct contact between intestinal and luminal pathogens and toxins, thus promoting intestinal inflammation [77]. In addition, studies have shown that intestinal mucosal mechanical barrier injury can greatly increase IBD risk and colorectal cancer in mice, revealing the importance of the intestinal mucosal mechanical barrier in regulating inflammation and tumor process [78]. Current studies have found that the claudin family of intestinal mucosal mechanical barrier compact proteins is associated with different types of tumors, including breast cancer, prostate cancer, ovarian cancer, pancreatic cancer, gastric cancer, and colorectal cancer [79–81]. They may provide a signal pathway that connects the inside and outside of the cell and induces the proliferation and migration of cancer cells [82]. Mees et al. detected the content of tight junction proteins in the colon tissue of colorectal cancer patients with a history of UC and found that the expression of Claudin1,3,4 and *β*-catenin in patient tissue was significantly higher than that in healthy tissue [83]. Garcia-Hernandez et al. [84] found that when mucosal inflammation occurred, the expression of claudin-1, -2, and-18 in the intestinal epithelium increased, while the expression of claudin-3, -4, -5, -7, -8, and-12 decreased. The destruction of tight junction proteins in IBD tissue can often reflect the severity of inflammation and the prognosis of patients to a certain extent, and inflammation is also an important risk factor affecting the progression of inflammatory bowel disease and colorectal cancer. Many studies have found that lncRNAs can participate in the development of colorectal cancer by regulating claudin protein in the intestinal mucosal mechanical barrier. The lncRNA CCAT-1 mentioned earlier in this paper is closely related to the occurrence and development of CRC. It has been found that in patients with colorectal cancer, CCAT1 can modulate MLCK in a miR-185-3p-dependent manner, regulate the role of tight junction proteins including claudin and ZO-1 in the distribution of MLCK, increase intestinal epithelial TJ permeability, and promote the malignant change of IBD [43]. lncRNA SPRY4-IT1 showed a similar effect. Some studies have suggested that it inhibits the translation of claudin-1, claudin-3, jam-1, and occludin in intestinal barrier tight junction proteins, reduce their stability, and lead to intestinal mechanical barrier dysfunction and promote the progression of colorectal cancer [59]. The lncRNA UC.173 plays the opposite role. lncRNA UC.173 can act as a molecular sponge of miR-29b that specifically binds to it, reducing its inhibitory effect on CLDN1 mRNA, promoting the expression of claudin-1, and indirectly repairing the damaged intestinal mucosal machinery barrier function to improve the symptoms of colorectal cancer patients [62].

### 4.3. lncRNA Participates in the Occurrence and Development of IBS-D by Regulating Intestinal Mucosal Mechanical Barrier

Irritable bowel syndrome with diarrhea (IBS-D) is the most common subtype of IBS. Patients often experience greater mental stress and psychological problems to a certain extent [85]. The intestinal permeability of IBS-D patients is often elevated, which is considered to be one of the causes of diarrhea [86]. In recent years, a growing number of studies have discovered that IBS-D occurrence is related to the intestinal mucosal mechanical barrier. An animal experiment confirmed that intestinal permeability was significantly increased in IBS-D mice [87]. Chao and Zhang's study also confirmed that IBS-D is caused by increased intestinal mucosal permeability, which could be related to the low expression of AQP 1, 3, 8 [88]. In particular, there is a significant correlation between the reduction in AQP3 and diarrhea symptoms [89]. Aquaporin exists mainly in human intestinal epithelial cells and plays an important role in maintaining the normal function of the intestinal tract [90]. We previously detected the expression levels of lncRNAH19, AQP1, and AQP3 in the colon of patients with IBS-D; we found that their expressions were significantly downregulated; we then demonstrated that their reduction is proportional through cell experiments. This suggests that the downregulation of lncRNA H19 affects the expression of AQP1 and AQP3, enhances the permeability of the intestinal mucosal mechanical barrier, and may be involved in IBS-D development [32].

### 4.4. lncRNA Participates in the Progression of Celiac Disease by Regulating the Homeostasis of Intestinal Mucosal Mechanical Barrier

Celiac disease, also known as gluten allergy, is an autoimmune digestive tract disease typically characterized by intestinal inflammation and intestinal mucosal damage [91]. Castellanos-Rubio et al. [92] reported a lncRNA, lnc13, which contains a haplotype block associated with celiac disease and inhibits the expression of certain inflammatory genes under steady-state conditions. Lnc13 regulates gene expression by binding to hnRNPD, a member of the widely expressed heterogeneous ribonucleoprotein (HnRNP) family. The level of lnc13 decreased under stimulation, allowing the expression of inflammatory genes to increase. The authors believe that lnc13 plays an important role in maintaining intestinal mucosal barrier function, and the downregulation of lnc13 expression leads to the impairment of intestinal mucosal barrier function and an increase in intestinal barrier permeability. The level of LNC13 in small intestinal biopsies from patients with celiac disease was significantly decreased, suggesting that the downregulated expression of LNC13 may be one of the causes of inflammation in celiac disease. It has also been found that the noncoding regions of celiac disease-related SNPs can produce long noncoding RNAs (lncRNAs), many of which are regulators of gene expression. Many disease-related SNPs located in lncRNAs change their secondary structures or affect their expression levels, thus affecting their regulatory function, destroying the homeostasis of the intestinal mucosal barrier, thus promoting the development of the disease [93]. Recently, Santin et al. [94] reported a lncRNA with a new extraceliac risk variant named HCG14, which can regulate the expression of NOD1 in an allele-specific manner. NOD1, a member of the NOD-like receptor (NLR) family, is one of the most studied pathogen recognition receptors (PRRs). It acts as the first barrier against pathogens in several other tissues, including the intestinal tract. However, the mechanism underlying the increased risk of CD caused by HCG14 is still unknown and needs to be further explored.

## 5. lncRNA as a New Diagnostic and Therapeutic Marker of Inflammatory Bowel Disease

### 5.1. lncRNA as a New Diagnostic Marker of Inflammatory Bowel Disease

At present, IBD diagnosis lacks convincing gold standards. Routine diagnosis of IBD includes clinical symptom assessment combined with endoscopy, histology, serology, and radiology [95]. At the same time, IBD lacks specific biomarkers, often leading to misdiagnosis and delayed treatment. lncRNAs are a valuable diagnostic marker for various diseases. Its abundance in vivo is high and can be quickly detected by common molecular biological techniques, and has relative stability and tissue specificity [96]. Currently, many studies on IBD, colon biopsy, and lncRNA map data from blood samples suggest significant differences between the disease group and the healthy group [97]. By detecting lncRNAs, we can classify IBD subtypes and determine whether IBD is active. It is hoped that noninvasive lncRNAs based on humoral fluid can be used as biomarkers [98]. This utility improves our ability to diagnose IBD greatly and enables us to predict the occurrence of IBD before its clinical symptoms appear. In addition, because the pathological process of IBD is very complex, a single lncRNA corresponding to a certain stage is not sufficient to meet the needs of clinical diagnosis. Therefore, the combination of several candidate lncRNAs from different tissue sources and available biomarkers may be necessary to provide an accurate diagnosis. Overall, we can increase the likelihood of introducing reliable lncRNAs as IBD biomarkers through a larger cohort study of tissue biopsies and body fluids.

### 5.2. lncRNA as a Potential Therapeutic Target for Inflammatory Bowel Disease

lncRNAs are potential therapeutic targets for IBD. Downregulation of lncRNA by RNA interference or forced overexpression of lncRNA by appropriate vectors may affect the IBD process [99]. Although initial successes in treating intestinal diseases based on lncRNAs have been made in animal studies, these methods have not been proven feasible and safe in the clinic. Currently, immunosuppressant or hormone therapy is the main treatment for IBD [100]. Recent studies have shown that repairing the intestinal mucosal mechanical barrier can induce continuous clinical remission in patients with IBD, reduce the number of hospitalizations and operations, and improve the quality of life of patients [101]. Therefore, repairing the mechanical barrier of the damaged intestinal mucosa by artificially interfering with the expression of lncRNA has become an important research direction in the treatment of IBD. In recent years, there has been a new understanding of the role of lncRNAs in the inflammatory mechanism of IBD [102]. However, little is known about the key regulators that activate, fine-tune, or turn off NF*κ*B activity under inflammatory conditions. Akıncılar et al. [103] designed the first genetic screening method to identify the specific lncRNA of NF*κ*B and found a conservative lncRNA named NAIL. After a series of experiments, the authors found that NAIL can cooperate with another inflammatory factor, P38, to activate NF-*κ*B and induce progenitor cells to differentiate into immature myeloid cells in the bone marrow, macrophages reassemble to the inflammatory region, and express inflammatory genes in colitis. Inactivated lncRNA NAIL can reduce the inflammatory response in colitis mice, suggesting that NAIL is an ideal target and biomarker for treating inflammatory bowel disease and other inflammation-related diseases.

### 5.3. lncRNA Can Be Used as a Marker for the Diagnosis and Treatment of Other Intestinal Diseases

In addition to IBD, lncRNAs can also be used as markers for diagnosing and treating other intestinal diseases such as colorectal cancer and celiac disease (Table 2). In the pathogenesis of colorectal cancer, many lncRNAs compete with specific mRNAs in binding to miRNAs. These lncRNA-miRNA-mRNA competitive endogenous RNA networks form a complex and highly regulated mechanism to control gene expression and cell function [104]. lncRNA members of this network are often involved in the advanced stage of colorectal cancer (such as CACS15, CYTOR, HOTAIR, MALAT1, TUG1, NEAT1, and MIR17HG) [105]. These lncRNAs may be effective prognostic biomarkers. More importantly, the knockout or overexpression of these members in the colorectal cancer-related Cerna network significantly inhibits colorectal cancer progression, indicating their potential as therapeutic targets for colorectal cancer. In celiac disease, researchers propose that increasing the content of lnc13 can inhibit the expression of inflammatory genes associated with celiac disease, revealing the potential of lnc13 as a potential target for the diagnosis and treatment of celiac disease [91].

## 6. Summary and Outlook

The intestinal mucosal mechanical barrier is the most important barrier to prevent trillions of microorganisms, foreign antigens, and viruses from entering the intestinal environment. Its existence maintains the delicate dynamic balance between intestinal microorganisms and host epithelial cells, and plays a very important role in preventing intestinal mucosal damage. Damage to the intestinal mucosal mechanical barrier often leads to intestinal disease. As a rising star molecule in biology, lncRNAs have been shown to regulate various physiological and pathological processes. With the continuous progress of high-throughput sequencing technology [106], a growing number of lncRNAs have been annotated, but the functions of most lncRNAs remain unclear. Therefore, the study of lncRNAs is a broad unknown territory, which is of great research value and significance. In recent years, an increasing number of studies have revealed that lncRNAs have a regulatory effect on the intestinal mucosal mechanical barrier, and an increasing number of regulatory mechanisms are being found [107]. For example, various lncRNAs, including H19, regulate the function of intestinal epithelial cells and destroy the tight junctions between cells, resulting in an abnormal increase in the permeability of the intestinal mucosal mechanical barrier and affecting the normal function of the intestinal barrier. At the same time, injury to the intestinal mucosal mechanical barrier often leads to intestinal inflammatory diseases. A recent study found that the enhancement of intestinal mucosal mechanical barrier permeability is an important precursor of intestinal changes in patients with IBD and often occurs several years earlier. Presently, there is a lack of a gold standard for the diagnosis and treatment of IBD, which makes it urgent to find a specific marker for IBD diagnosis and treatment. Due to the gradual progress of high-throughput detection methods for lncRNA, the difficulty of detection and intervention of specific lncRNAs is significantly reduced, greatly improving the possibility of lncRNA becoming a new diagnostic and therapeutic target for IBD. Recently, there has been a breakthrough in studying the mechanism of inflammation caused by lncRNAs. Some studies [85] have found that lncRNAs play a very significant role in the process of activating intestinal inflammatory genes. We have every reason to believe that the diagnosis and treatment of intestinal diseases will have a broad and bright prospect in the near future through specific detection and monitoring of the corresponding lncRNA.

## Figures and Tables

**Table 1:** Regulatory effect and mechanism of different lncRNAs on intestinal mucosal mechanical barrier.

lncRNA	Impact on the barrier	Regulation methods	Action object	Functions
H19	↓	Direct	Paneth cell and goblet cell	Promote autophagy of small intestinal mucosa [25].
	↓	Indirect	miR-675	Inhibit the expression of TJ ZO-1 and E-cadherin, resulting in epithelial barrier dysfunction [27].

H19	↑	Indirect	miRNA LET-7G	Promote the repair of intestinal epithelial mucosa after burn [29].
	↑	Direct	AQP1, AQP3	Promote the expression of AQP and maintain the stability of intestinal mucosal mechanical barrier [32].
	↑	Indirect	P53, miRNA34a, let-7	Promote IECs proliferation and epithelial regeneration [33].
	↑	Indirect	miR-675-5p	Intestinal mucosal barrier damage caused by inhibition of VDR expression [36].

CCAT1	↓	Indirect	miR-185-3p	Increase the permeability of intestinal barrier and destroy the function of intestinal barrier [43].

PlncRNA1	↑	Direct	MAZ, ZO-1, occludin	Significantly enhance the protective function of intestinal barrier against sodium sulfate paste (DSS) injury [48].

neat1	↓	Direct	IEC macrophages	Participate in inflammatory response by regulating intestinal epithelial barrier and exocrine-mediated macrophage polarization [52].

SPRY4-IT1	↑	Direct	TJ	Change the expression of tight junction (TJ) protein to enhance the function of intestinal epithelial barrier [59].

uc.173	↑	Indirect	miRNA195 miR-29b	Promote the translation of TJ claudin-1 (CLDN1) and the repairment of intestinal mucosal mechanical barrier [61].

Bmp1	↑	Indirect	miR-128-3p	Increase the proliferation and migration of IEC-6 or HIEC-6 cells in rat intestinal crypt epithelial cells and promote the repair of intestinal mucosal mechanical barrier [65].

BC012900	↓	Direct	IECs	Inhibit the proliferation of intestinal epithelial cells and increase the sensitivity of cells to apoptosis [66].

**Table 2:** Summary and mechanism of lncRNA as a marker for diagnosis and treatment of various intestinal diseases.

Intestinal disease	Related lncRNA	Regulating mechanism	A potential role as a marker of diagnosis or treatment
IBD	CNN3-206	The lncRNA CNN3-206-miR-212-Caspase10 regulatory network	In intestinal lesions of patients with Crohn's disease, the expression of lncRNA CNN3-206 is significantly increased [74].
	CRNDE	miR-495 and SOCS1	Indirectly induce apoptosis of intestinal mucosal epithelial cells and aggravate the inflammatory response of IBD, can be used as a potential therapeutic target [75].
	NAIL	p38 and NF*κ*B	Targeted knockout of NAIL can inhibit the expression of downstream inflammatory factors and greatly reduce the intestinal inflammatory response in patients with IBD [102].

Colorectal cancer	CCAT-1	miR-185-3p Claudin, ZO-1	In patients with colorectal cancer, the expression of CCAT is significantly increased, and the intestinal barrier function can be significantly improved by inhibiting its expression [42].
	SPRY4-IT1	Claudin-1, claudin-3, occludin, and jam-1	SPRY4-IT1 can destroy intestinal TJ and cause intestinal epithelial barrier dysfunction, which can be used as a potential therapeutic target [84].
	UC.173	miR-29b	Promoting the expression of uc.173 can advance the translation of TJ, claudin-1 (CLDN1), promotes the repair of intestinal mucosal mechanical barrier, and is beneficial to the improvement of symptoms in patients with colorectal cancer [61].

IBS-D	H19	AQP 1, 3, 8	Inhibiting H19 expression can significantly promote AQP1, AQP3, and AQP8 expression and significantly improve intestinal barrier function in IBS-D mice [87].

Celiac disease	Lnc13	hnRNPD	The expression of LNC13 in intestinal biopsies of patients with celiac disease was significantly decreased, suggesting that the downregulated expression of LNC13 may be one of the causes of inflammation of celiac disease [91].
	HCG14	NOD1	The content of HCG14 in intestinal tract of patients with celiac disease increased significantly, suggesting its potential value as a diagnostic index of celiac disease [93].
